# Plant probiotic bacteria enhance the quality of fruit and horticultural crops

**DOI:** 10.3934/microbiol.2017.3.483

**Published:** 2017-06-19

**Authors:** Alejandro Jiménez-Gómez, Lorena Celador-Lera, María Fradejas-Bayón, Raúl Rivas

**Affiliations:** 1 Department of Microbiology and Genetics, University of Salamanca, 37007 Salamanca, Spain; 2 Spanish-Portuguese Institute for Agricultural Research (CIALE), Spain; 3 Associated I + D Unit, USAL-CSIC (IRNASA), Salamanca, Spain

**Keywords:** bacterial inoculants, food quality, plant growth promoting bacteria, biofertilizers, green commercial products

## Abstract

The negative effects on the environment and human health caused by the current farming systems based on the overuse of chemical fertilizers have been reported in many studies. By contrast, bacterial inoculations produce positive effects on yields without causing this type of harm. Hence, during recent years, the commercialization of biofertilizers has been on the increase, and the number of companies and products available are expanding worldwide every year. In addition to the notable enhancement of crop production, many studies have shown how the application of bacteria has positive effects on food quality such as improved vitamin, flavonoid and antioxidant content, among other benefits. This advantage is interesting with respect to food that is consumed raw, such as fruits and many vegetables, as these bioactive molecules are maintained up until the moment the food is consumed. As regards this review focuses on the collection of studies that demonstrate that microorganisms can act as plant probiotics of fruit and horticultural crops, essential types of food that form part of a healthy diet.

## 1. Introduction

Modern farming systems used worldwide to satisfy the increasing demand for food require the extensive use of chemical fertilizers, which are not only very costly but produce many environmental and human health problems [1]. Also, consumers have become more aware of different food production systems and food safety and quality [2]. In recent years, new production methods to ensure food quality have been investigated, addressing a key area within the European political agenda [3]. Due to general environmental awareness, these new methods are often in line with the ideology of green politics, and many countries have implemented these methods and have included them within their legislation.

Currently, there are many studies that have analysed increased crop production following the use of several bacterial strains. And, there is also a significant number of studies that not only show increased total yield productions, but also the nutritional benefits achieved by the application of biofertilizers on plants and the role of bacteria in improving the quality of crops [4].

Recently, the plant-associated microorganisms which cause beneficial effects have been defined as “Plant Probiotic” [5],[6]. Plant probiotics are microorganisms that confer benefits to the health of plants when administered in specific amounts [7]. In addition, they belong to the bacterial group “Plant Growth Promoting Rhizobacteria” (PGPR) which are able to colonize plant roots [8],[9], promote plant growth through different direct and indirect mechanisms [1],[10], improve plant nutritional content [11],[12] and increase crop quality [13],[14].

However, it is known that in order to achieve benefits to plants, the selection of efficient and specific PGPR is required. Therefore, the complex interactions between plants, bacteria and environmental factors need to be understood. Environment factors such as inorganic mineral content and plant exudates are associated with the growth and establishment of bacteria [15],[16]. Thus, the microbial communities associated with plants depend on ecosystem development [16]. Understanding how microbial communities colonize plant roots [17] and the interactions and mechanisms of biofertilizers is essential if plant quality is to be improved [18]. Another key factor is to ensure the non-pathogenicity of the microorganisms used in biofertilization processes, with the aim to avoid problems to human health or the environment [19].

Among current food guidelines, one of the new initiatives is based on the consumption of vegetables and fruits rich in bioactive substances. These substances are able to express anticarcinogenic effects and prevent various diseases and health disorders [20]. Consequently, the beneficial effects of bioactive substances on human health have been analysed in several studies [5],[21]. Moreover, the high consumption of these types of foods is recommended for a healthy diet. For example, berries provide significant health benefits owing to the high level of vitamins, minerals and antioxidants they contain [20]. In addition, berries are readily available and can be consumed in a wide range of forms, such as fresh or frozen, and can be found in food products like jam and yogurt [22]. On the other hand, horticultural crops provide humans with a large variety of indispensable vitamins, such as A and C, and elements such as potassium [23]. According to Ramsay et al. [24], consumption of a variety of fruits and vegetables is linked to overall diet quality.

This review focuses on improved crop quality after the application of plant probiotic bacteria as enhancers of the nutritional value of fruits and horticultural crops.

## 2. Impact of Bacterial Probiotics on Plant Nutrient Content

Vitamins are among the nutrients required for many physiological functions essential to life. Despite being presented in minute amounts on food, vitamins prevent specific deficiency syndromes which can affect people when there is an absence or a reduction of their contents [25]. Moreover, vitamin deficiency in humans can produce several diseases such as ocular surface abnormalities [26] or neurodegenerative problems [27]. Due to the importance of vitamins, one of the proposals presented by the World Health Organization (WHO) is to improve the content of essential vitamins in food in order to decrease worldwide malnutrition [28]. Therefore, scientists are searching for different ways to increase the total content of these types of substances in crops. Here, some of the studies in which bacterial plant probiotic inoculation has been used to increase the vitamin content of fruits and horticultural crops (Table 1) are presented.

Vitamin C is the vitamin with the highest recommended daily dose and L-Ascorbic acid (AsA) is derived from L-threo-hex-2-enono-1,4-lactone. Some mammals cannot synthesize AsA due to the lack of functionality of the gene that encodes the enzyme which catalyses its biosynthesis [29]. The results obtained by Flores-Félix et al. [5] showed that vitamin C levels were significantly higher (79% compare to the control) in strawberries obtained from plants inoculated with the strain *Phyllobacterium* sp. PEPV15. Moreover, Pirlak et al. [30] compare the results obtained, depending on the type of inoculation, and report that the highest levels of vitamin C in strawberry were obtained by applying root and foliar application in comparison with the uninoculated treatment were higher. The strains used in these experiments were *Pseudomonas* BA-8, *Bacillus* OSU-142 and *Bacillus* M-3. Erturk et al. [31] also showed high levels of vitamin C content in strawberry fruits after plants were inoculated with *Paenibacillus polymyxa* RC05.

Increased levels of vitamin C after bacterial treatment have also been described for vegetables. For instance, Bona et al. [30] shows that inoculation with the strain *Pseudomonas* sp. 19Fv1T not only enhances yield but also positively affects the concentration of vitamin C in tomato fruits in comparison with the control treatment. Additionally, Shen et al. [33] shows that vermicompost combined with plant probiotic *Bacillus megaterium* and *Bacillus amyloliquefaciens* also increases tomato yield and vitamin C contents. According to Gül et al. [34], the highest levels of vitamin C content in tomato fruits were obtained after the inoculation of two bacterial strains *Bacillus amyloliquefaciens* (FZB2 and FZB42) in different treatments.

Berry crops are regarded as a good source of vitamins in addition to their anticarcinogenic and antimutagenic properties [22],[35]. In this sense, Bona et al. [36] analyses the improvement of vitamin B9 and vitamin C content in strawberry fruits after the inoculation with arbuscular mycorrhizal fungi (AMF) and different strains of plant growth promoting bacteria (PGPB). They showed significant differences in ascorbic acid levels compared to the control after the inoculation with *Pseudomonas* sp. 5Vm1K, a mixture of AMF, and a co-inoculation formed by AMF and *Pseudomonas* sp. 5Vm1K. However, the vitamin B9 content was only significant higher after double co-inoculation with AMF and PGPB.

Moreover, in the literature it is known that some vitamins also have antioxidants effects, being substances that interfere with normal oxidation processes, such as oxygen scavengers, promoting a more favourable anaerobic environment [37]. Thus, plant antioxidants are one of the most active food compounds [38] and are present in vegetables like tomato, carrot, cabbage, red beets and fruits as cocoa, prunes, red grapes and citrus fruits [39]. These substances can be classified into two different groups according to their mode of action: chemical substances which interrupt free radical chain propagation, and oxygen scavengers and chelators which bind to ions involved in the formation of free radicals [39].

Here, we present some of the studies published on fruit and horticultural crops that have shown a significant increase in antioxidant activity after being inoculated with plant probiotic bacteria (Table 1).

Tomato, one of the most cultivate vegetables in the world, is among the vegetables and horticultural used in these types of studies [40]. Moreover, tomatoes are regarded as an excellent source of antioxidant compounds [32],[41]. Ochoa-Velasco et al. [14] compared the different levels of total antioxidant content in tomato fruits after biofertilization with *Bacillus licheniformis* and different doses of a nitrogen fertilizer. In general terms, they describe an improvement in the quality of tomato fruits with respect to total hydrophilic antioxidant compounds (vitamin C and total phenols) after inoculation with *B. licheniformes* and the reduction of the nitrogen doses.

The bacterial effects on antioxidant activity have also been analysed in basil (*Ocimum basilicum* L.). This medicinal plant is used worldwide in cooking, and its antioxidant activity has been investigated after different treatments. The inoculation of basil with a mixture of *Pseudomonas putida* 41, *Azotobacter chroococcum* 5, *Azospirillum lipoferum* OF strains presented the highest level of antioxidant activity [42] as compared to the control treatment. In addition, another study involving basil reported that, under water stress, inoculation with three bacterial species (*Pseudomonas* sp., *Bacillus lentus* and *Azospirillum brasilens*) also increased antioxidant activity (Catalase and guaiacol peroxidise activity), as well as a significant change in leaf chlorophyll content [43].

Peppers (*Capsicum annuum* L.) are also considered as an excellent dietary source of antioxidant compounds, in addition to being an economically important agricultural crop. In the study published by Silva et al. [44], pepper extracts were tested against 1,1-Diphenyl-2-picrylhydrazy, superoxide (O2 ^•−^), and nitric oxide (^•^ NO) radicals. The results showed a significant difference between the extract from the fruits of inoculated plants and the extracts from uninoculated plants. Also, higher antioxidant activity was found in peppers from plants inoculated with the strain *Rhizobium leguminosarum* PETP01.

Antioxidant activity has also been analysed in fruits. And, strawberries have been found to be a good source of antioxidants. The anthocyanin content is in part responsible for the antioxidant power in berries [45]. Lingua et al. [46] reported that the co-inoculation of plant growth promoting bacterial strains and mycorrhizal fungi on strawberry plants produced the best levels of anthocyanin content in strawberries.

Carotenoids are natural pigments which belong to the second group of antioxidants. They are involved in indispensable roles in photoprotection, stress and photosynthesis, in addition to acting as precursors of vitamin A [47],[48]. Some carotenoids compounds have been reported to prevent humans from developing certain cancers and cardiovascular diseases. These compounds also protect plants against photooxidative damage and contribute to maintaining the plants overall structure.

Lycopene is a red carotenoid that is an intermediate in the biosynthesis of other carotenoids, such as β-carotene [49], and is an effective free radical scavenger. Also, its presence in the human diet is correlated with a reduction in the incidence of cancer [3],[50]. Lycopene is involved in the reddening of the tomato due to its function in the differentiation of chloroplasts and chromoplasts. In addition, lycopene is important in the final nutritional quality of this plant product [51]. With regard to the importance of carotenoids, during the past years scientists have being analyzing the way to improve their contents in crops by using fertilizers based on bacterial plant probiotics. Ordookhani et al. [52] concluded that the inoculation of tomato plants with *Pseudomonas putida* 41, *Azotobacter chroococcum* 5, *Azospirillum lipoferum* OF and a mixture of arbuscular mycorrhiza fungi *(Glomus lipoferum, Glomus mossea* and *Glomus etunicatum)* increased lycopene antioxidant levels. Moreover a positive correlation between lycopene and fruit potassium content was found in tomato plants after inoculation.

In order to increase crop yield, large amounts of N and P are required and excessive amounts are applied to the soil, causing significant environmental pollution as a result of nitrate leaching [53]. To reduce these adverse effects, PGPR bacteria can be used as an alternative to chemical fertilizers as they can improve plant growth through plant nutrient uptake and thus improving crop production [54].

PGPRs have direct mechanisms such as nitrogen fixation, phosphate solubilization, phytohormones production, among others, that facilitate the availability and absorption of nutrients [55],[56].

Plant growth promoting bacteria are used in numerous studies which also have such mechanisms as nitrogen fixing bacteria [57]. Consequently, the increase of N content may produce a greater uptake of nutrients present in the soil by the plant and an increase of the plant nutrient element (PNE) contents, improving the quality of the crops [58].

Many studies use PGPR microorganisms to improve the quality of various crops by increasing the PNE content. For example, the increase in Ca content produces a more rigid peel when fruit is ripe. On the other hand, an increase in K content leads to an increase in the translocation of carbohydrates from the leaves to the fruit and, consequently, a higher fruit volume is obtained [59]. Some studies have evaluated the nutritional content of fruits and crops after inoculation with PGPR bacteria.

Broccoli is a vegetable rich in dietary fiber, nutrients and several phytochemicals with proposed health-related properties [11]. Yildirim et al. [58] inoculated broccoli roots with the PGPR bacteria (*Bacillus cereus*, *Brevibacillus reuszeri* and *Rhizobium rubi*) to evaluate if they had any additional effect on crop growth and nutrient uptake. The results obtained from the inoculated plants showed an increase in chlorophyll, N, K, Ca, S, P, Mg, Fe, Mn, Zn and Cu contents. In addition, crop yield increased by 17.0%, 20.2% and 24.3% and the chlorophyll content by 14.7%, 14.0% and 13.7% in treatments inoculated with *Bacillus cereus, Rhizobium rubi* and *Brevibacillus reuszeri*, respectively.

As mentioned above, tomato fruit is one of the most consumed vegetables in the world, either fresh or processed [60]. Sharafzadeh [61] performed a greenhouse test, inoculating tomato plants with different combinations of PGPRs such as *Pseudomonas, Azotobacter* and *Azosprillum.* The result obtained from 5 out of the 7 inoculated treatments showed an increase in the nutrient content of N, P, K, Ca and Mg.

Vayssières et al. [62] obtained similar yields of banana crops in two experiments: a treatment with a nitrogen fertilizer (100%) and another treatment involving inoculation with PGPB and lower nitrogen fertilizer conditions (33%). The results showed that the inoculations with *Azospirillum brasilense* Sp7 and *Bacillus sphericus* UPMB10 improved fruit yield and fruit quality under the reduced fertilizer conditions. The nutrients which were shown to be increased in the treatments with the PGPB were N, P, K, Ca and Mg. Thus, it was concluded that these strains could be used as biofertilizers to improve banana production and quality.

**Table 1.** Plant probiotic bacteria used to enhance the quality of fruit and horticultural crops.

Bacterial strain	Fruit or horticultural crop	Crop effects caused	Reference
*Phyllobacterium* sp. PEPV15	*Fragraria x ananassa*	Enhancement of vitamin C	[5]
*Pseudomonas* BA-8, *Bacillus* OSU-142 and *Bacillus* M-3	*Fragraria x ananassa*	Enhancement of vitamin C	[30]
*Paenibacillus polymyxa*	*Fragraria x ananassa*	Enhancement of vitamin C	[31]
*Pseudomonas sp.* 19Fv1T	*Lycopersicon esculentum*	Enhancement of vitamin C	[32]
*Bacillus megaterium* and *Bacillus amyloliquefaciens*	*Lycopersicon esculentum*	Enhancement of vitamin C	[33]
*Bacillus amyloliquefaciens* FZB2 and *B. amyloliquefaciens* FZB42	*Lycopersicon esculentum*	Enhancement of vitamin C	[34]
*Pseudomonas sp.* 5Vm1K and AMF	*Fragaria × ananassa*	Enhancement of vitamin B and C	[36]
*Pseudomonas putida* 41, *Azotobacter chroococcum*5, *Azospirillum lipoferum* OF and AMF	*Lycopersicon esculentum*	Increased antioxidant activity	[52]
*Bacillus licheniformis*	*Lycopersicon esculentum*	Improved total flavonoids content	[14]
*Pseudomonas putida* 41, *Azotobacterchroococcum*5, *Azospirillum lipoferum* OF	*Ocimum basilicum*	Increased antioxidant activity	[42]
*Pseudomonas sp., Bacillus lentus* and *Azospirillum brasilens.*	*Ocimum basilicum*	Increased antioxidant activity and chlorophyll leaf content	[43]
*Rhizobium leguminosarum* PETP01	*Capsicum annuum*	Increased antioxidant activity	[44]
*Pseudomonas fluorescens* N21.4	*Rubus sp.*	Increased flavonoids concentration	[13]
*Rhizobium* sp. PEPV12	*Spinacia oleracea.*	Increase chlorophyll content	[9]
*Paenibacillus polymyxa* RC14	*Brassica oleracea* var capitata cv Yalova 1.	N, P, K, S, Fe, and Cu content increase	[12]
*Bacillus subtilis* BA-142, *Bacillus megaeorium*- GC subgroup A. MFD-2, *Acinetobacter baumannii* CD-1 and *Pantoea agglomerans* FF	*Lycopersicon esculentum* L. and *Cucumis sativus* L.	N, P, Mg, Ca, Na, K, Cu, Mn, Fe and Zn content increase in both fruit	[114]
*Bacillus subtilis* EY2, *Bacillus atrophaeus* EY6, *Bacillus spharicus* GC subgroup B EY30, *Staphylococcus kloosii* EY37, and *Kocuriaerythromyxa* EY43	*Fragaria x ananassa.*	N, P, K, Ca, Mg, S, Mn, Cu and Fe	[115]
*Pseudomonas fluorescens* and *Bradyrhizobium* sp.	*Origanum majorana*	Increase the amount of essentials oils	[65]
*Bacillus megaterium, Pantoea agglomerans* and *Bacillus subtilis*	*Brassica oleracea*	Increase chlorophyll plant content	[116]
*Pseudomonas fluorescens* N21.4	*Rubus sp.* var Lochness	Increase and stabilize total flavonoid content during different season	[79]

## 3. Effects of Plant Probiotics Bacteria on Flavonoids, Organic Acids and Volatile Compounds Levels

Plants usually produce organic compounds as root exudates. These compounds are usually metabolites from different metabolic routes such as flavonoids, organic acids, sterols and volatile compounds [63]. They have diverse functions in plants like providing protection against pathogens [64]–[67] or abiotic stresses [68], as well as increasing the nutrient content of plants [69] and therefore their quality. The concentration of these metabolites in plant tissues varies depending on their biosynthesis, storage and degradation processes [63]. Furthermore, some of these compounds can be beneficial to human health when consumed in large quantities, thus they are important not only for the food industry but also for pharmaceutics companies [70].

Flavonoids are the largest group of phenolic compounds that are abundantly present in plants, and are one of the major groups of plant secondary metabolites [71]. They are considered as having a wide range of biochemical and pharmacological properties. Recently, the analysis of flavonoid content in plants has received considerable attention due to fact that these substances possess biological properties, such as chemoprevention [72], and its role in the protection against diseases [73],[74]. In addition, they are known to present antifungical, antibacterial and antiviral effects [75]. Considering all of the beneficial effects of flavonoids on human health, several research projects have been mentioned in the literature where scientists attempt to enhance flavonoid content by bacterial inoculation of crops with special economic interest.

Since fruits are regarded as a good source of flavonoids, Basu and Maier [76] analysed the flavonoid content in different species of berries crops, showing that all of their antioxidant activity had the potential to improve human health. Blackberry is an important type of berry, which provides beneficial health effects [77]. García-Seco et al. [78] shows that the inoculation of blackberry plants with the strain *Pseudomonas fluorescens* N21.4 improves flavonoid content by 22%, as compared to the control treatment, during a specific period of the year (from July through October). Taking these results into account, Ramos-Solano et al. [79] analysed the effect of the bacterial probiotic inoculation on plant quality throughout the year and reports that the strain N21.4 tends to increase and stabilize total flavonoid content in blackberry crops, even when environmental conditions are adverse. Moreover, García-Seco et al. [13] reported that the blackberry root treatment with *Pseudomonas fluorescens* N21.4 not only increased the expression of flavonoids biosynthetic genes, but also flavonoid concentration in fruits. In the research published by Ghorbanpour et al. [80], several *Pseudomonas fluorescens* strains were also analysed with the intention of determining their role on plant growth and flavonoids production in other type of crops. They showed that the inoculation with the strain *Pseudomonas fluorescens* Ap14 induced a significantly higher flavonoid content in comparison with the uninoculated control treatment. Apart from enhancing the berries overall quality, bacterial probiotics have been reported to delay fungal growth [81].

Beneficial effects in horticultural crops have also been shown. Silva et al. [44] showed that aqueous extracts of *Capsicum annuum* leaves, after the bacterial inoculation with the *rhizobium* strain TVP08, presented a significant acetylcholinesterease (AchE) inhibitory activity (with an important relevance in the treatment of Alzheimer's disease). This result could be explained by the presence of flavonoids.

Moreover, improved total flavonoid content has been described in fruit and horticultural species, and also in other types of crops such as buckwheat (*Fagopyrum esculentum* Moench). Singh et al. [82] showed that buckwheat inoculated with *Azospirillum* spp. and *Azotobacter* spp. produces increased concentrations of flavonoid and phenolic contents.

Organic acids play an important role in plant responses to nutrient stress, and their content in plants depends of their nutritional status [83]. Organic acids (glutamic, tartaric, quinic, malonic, malic, shikimic, a-ketoglutaric, pyruvic, citric, succinic and fumaric acids) are widely distributed in fruits (melon, grape, peach, orange, lemon) and vegetables (green and red pepper, tomato, lettuce and lamb's lettuce) and influence the nutritional state, quality food and organoleptic properties like aroma, color and flavor [69].

Typically, roots exudates contain many organic acids such as acetate, malate, aconitate, lactate, fumarate, isocitrate, oxalate, citrate and succinate. These compounds are the primary anion components and make up a complex mixture of metal cations in solution; this in turn allows for the displacement of anions from the soil matrix and the mobilization of micronutrients like Zn and Cu into the rhizosphere [84].

Citrate and malate are involved in nutrient acquisition, and form potent complexes with Fe in soil and induce the dissolution of unavailable insoluble ferric oxyhydroxides. Moreover, they are the primary components released into the rhizosphere under P deficiency [84]. It has been shown that some PGPR can promote plant growth by capturing nutrients, since these bacteria produce an increase of organic acids in the roots of plants. For example, in strawberry crops the application of *Bacillus* and *Pseudomonas* strains produce an increase in plant biomass and nutrient content by the production of organic acids from bacteria [85], and in the same way in raspberry by the application of *Bacillus* [86], and in tomato, cucumber and sweet pepper by the application of *Pseudomonas* [87].

Fatty acids are among the organic compounds produced by root exudates and these compounds are mainly used for energy storage [88]. Some of them are not produced by the human body and must be introduced into the diet by eating vegetables (potato, cabbages, wheal and chive) and fruits (lemon, strawberry and orange) [89]. The importance of consuming these compounds is based on their biological properties which has been shown to have positive effects against cardiovascular diseases, arthritis and triglyceride levels. Cucurbit, strawberry and tomato are a good source of bioactive compounds and have an excellent nutritional composition, containing fatty acids among other compounds with antioxidants effects [90]. Some plant oils commonly used for human consumption have unsaturated fatty acids like linolenic, linoleic and oleic acid and saturated ones such us palmitic and stearic [88],[91]. Also, pumpkin seed oil has high nutritional value due to containing fatty acids like oleic, linolenid, palmitic and stearic. The application of phosphate solubilizing bacteria (*Pseudomonas putida* and *Bacillus lentus*) and nitrogen fixing bacteria (*Azotobacter sp.* and *Azospirillum sp.*) in pumpkin plants has been lead to an increase in oil, seed and fruit yield, especially the fatty acid content [92].

On the other hand, plants sterols are essential bioactive components of cell membranes which are present in high concentrations in vegetable oils [93]. Furthermore, the presence of these organic compounds in plants is related to protective effects against abiotic stresses [68]. These organic compounds are usually released in plant root exudates; the most abundant are campestrol, stigmasterol and sitoesterol and are found in the fruits and vegetables that make up the human diet [94]. The consumption of plants sterols may have some health benefits, like reducing the risk of cardiovascular diseases and protecting against different types of cancer [91]. It has been reported in the literature that the application of PGPR can produce an increment in the levels of sterols in plants. Silva et al. [95] inoculated two strains of *Rhizobium* (TVP08 and PEPT01) in pepper (*Capsicum annuum* L) and evaluated their effect on sterols. The inoculation of *Rhizobium* produced a positive effect on the ripening of the pepper fruit, in addition to an improvement in several primary and secondary metabolites, which improved the nutritional value of the plant.

Other organic substances which are common components of plants are volatile compounds. These can be found in small amounts in plants and are related to flowers, fruits, leaves, roots and vegetative tissues [96]. These compounds can act as a plant defense response against pathogens and predators [66]. Plants have control mechanisms that regulate the substances and the moment of the volatiles compounds synthesis. Their production can be influenced by the interaction with environmental microorganisms [96]. The application of rhizobacteria that promote plant growth can trigger an increase in the amount of volatile compounds, improving their qualities for the biological control of diseases [67]. In this respect, it has been shown that *Bacillus* [67],[97],[98] and *Pseudomonas* strains [90] can produce the inhibition of plant pathogens by the increment of volatile organic compounds in plants.

Essential oils are among the group of volatile compounds and are intensely aromatic compounds biosynthesized by plants [99]. It is known that soil microorganisms can modify the secondary metabolic routes of plants, influencing the synthesis of essential oils that are of great importance for the food and pharmaceutical industries [46]. The most volatile compounds contained in *Origanum majorana* L. are essential oils and have an important economic interest because of their use as flavoring, fragrances, fungicides and insecticides. Some authors have determined the effects of root colonization by PGPRs on the composition and amount of essential oils in different crops, and the inoculation of *Origanum majorana* with *P. fluorescens* and *Bradyrhizobium sp*. [65] and the inoculation of peppermint (*Mentha piperita*) with *P. ﬂuorescens* [100] produces an increase in the total essential oil content without modifying its composition.

Additionally, the production of antimicrobial compounds by plant probiotic bacteria should be highlighted. Many studies have reported that some PGPR strains are also able to produce these types of molecules, which have been shown to be effective against pest and pathogens and effective as biocontrol agents [101]. According to Zhen et al. [102], several *Bacillus* strains have an important effect on suppressing pathogens and promoting plant growth due to antagonistic relationships and the enhancement of the host's nutritional content and growth [103]. Antibiosis processes and an induction of systematic resistance (ISR) have also been reported to have effects on plant health [104]. The production of several organic acids, such as citric and ascorbic, which are strong antimicrobial agents, has been reported to be efficient at fighting against pathogens in vegetables and fruits due to a reduction in pH and anion accumulation [64]. Therefore, based on their mode of action and effects, many strains can also be used as biocontrol agents, which greatly enhance food quality and growth [102].

## 4. Situation, Characteristics and Problems Concerning the Current Commercialization of Plant Probiotic Bacterial Products

In addition to the many bacterial inoculants that have already been used to treat legume crops, the number of commercial products for other types of crops is increasing every year [1]. However the development and commercialization of new inoculants and formulas require governmental approval and must fulfill several requirements that are not always general and well established. Moreover, they depend on where the new product is registered [105].

In spite of being a worldwide process, one that is increasing year by year, there are no specific standards established for the creation and development of green products that can be used to reduce the amount of chemical fertilizers used, according to the new politics initiatives and programs (e.g. HORIZON 2020) [1]. Thus, the laws vary among countries and to date there is no international agreement regarding the quality control of biofertilizers [105]. Many countries have developed governmental policies for the use of biofertilizers, however, specifications to regulate such new products have not yet been established [3]. In this context, collaborations between researchers and industries are a key factor for establishing the basis of a worldwide biofertilizer market [1].

Suitable inoculants should be safe and nontoxic for environment and humans [106],[107],[108]. Herrmann et al. [109] shows that 64% of the commercial biofertilizers analyzed contained one or several contaminant strains, as well as human pathogens. Findings such as these highlight the need for better quality control of the inoculants that make up biofertilizers.

Moreover, in spite of the successful results so far achieved in the numerous studies involving plant growth promotion under controlled laboratories and greenhouses conditions, there have been variations within the results obtained in field trails. These differences can be explained by the abiotic and biotic conditions that can influence bacteria in the soil, as quite often bacterial inoculants are unable to survive in some specific niches [110]. Thus, according to Quin et al. [16] microbial plant communities fluctuate and depend on the state of the surrounding ecosystem.

Inoculant formulas should include the most effective plant growth promoting bacterial strains [111] and be designed to provide a high number of viable cells. Also, the presence of these strains should not become rapidly diminished [108]. The bacteria need to be able to survive in a broad spectrum of conditions, since crops are grown under a multiplicity of environmental and difficult conditions [107]. Thus, over the years, researchers have been working to develop new bacterial products active in a wide variety of weather conditions that can cause a difference in the benefits provided by the bacterial inoculants [112]. In this sense, according to Lesueur et al. [108], there are four types of products depending on the carrier material used: granules, liquids, slurries and powders. Herrmann and Lesueur [113] established the importance of a suitable carrier as a key factor to ensure bacterial protection against environmental conditions, microorganism survival and their establishment and action after the inoculation of crops.

Nowadays, despite all of the requirements and specifications, a large variety of bacterial inoculants, based on plant probiotic bacteria that enhances the quality of fruit and horticultural crops, are commercialized worldwide. This part of the review focuses on presenting some of the best known companies or sold products (Table 2). Nevertheless, the effects on food quality by the majority of commercialized bacterial products on sale today are not detailed or analyzed yet.

Due to the beneficial effects of fruit and vegetable crops on human health, the company “Guajart State Fertilizers & Chemical LTD” commercializes Azotobacter and Azospirillum culture®, a mixture of bacterial strains, to enhance the nutrient plant content in cash and horticultural crops.

Among the biofertilizers, useful for the production of horticultural crops such as tomatoes, Inogro® is produced and commercialized by the company “Flozyme Corporation” (USA). Inogro® is a mix of more than 30 bacterial species that not only enhance plant growth promotion and crop production, but also improves plant nutrient content.

One of the best results found in the improvement of tomato and pepper yields is achieved using Bonasol®, sold and produced by the company “Abiosa”. This product is based on a mixture of bacterial and arbuscular mycorrhizal fungi. Bonasol® improves phosphorus and potassium availability to the plants and enhances the plants' final nutritional state.

India is one of the countries whose government is making a large effort to try to increase the application of biofertilizers in their current farming system, and this has led to the development of companies such as Ajay Bio-tech (India) LDT. The company “Ajay Bio-tech (India) LTD” is one of the most important in India [105], and offers different quality enhancing products (Ajay Azo®, Ajay Azospirillum® and Ajay Meal®) used on many fruit and horticultural crops. These products are based on nitrogen-fixing or phosphate, and potassium-solubilising bacteria single or combined. Many of these strains enhance the uptake of indispensable nutrients by the plant, and overall plant nutrient content.

Thus, it is very important that the use of biofertilizers and the changes in traditional farming systems are approved by farmers worldwide. This is a key step for encouraging the commercialization and production of green products and will help to secure the foundations of a solid biofertilizer market [105].

**Table 2.** Commercial quality enhancing products used on fruit and horticultural crops.

Product	Company	Crop type	Effect caused
Pseudo-Guard Bio Fungicide ®	BioFix	Horticultural crops	Enhanced food quality
Ajay Azospirillum®	Ajay Bio-tech	Vegetables, cash and fruit crops	Enhanced chlorophyll content and enhanced vitamin, auxin, gibberellin and cytokinin production
Ajay Azo®	Ajay Bio-tech	Vegetable and horticulture crops	Enhanced auxin production
Ajay Meal®	Ajay Bio-tech	Fruit crops and vegetables	Enhanced overall quality
Inogro®	Flozyme corporation	Horticultural crops	Enhanced plant nutritional content
Azotobacter Culture®	Guajart State Fertilizers & Chemicals LTD	Banana, cash and horticultural crops	Enhanced plant nutritional content
Azospirillum Culture ®	Guajart State Fertilizers & Chemicals LTD	Banana, cash and horticultural crops	Enhance nutritional plant content
Prabha Zinc®	Prabhat Fertilizer & Chemical Works	Horticultural crops	Enhanced zinc content
Bonasol®	Abiosa	Tomato, pepper and chilli crops	Enhanced plant nutritional content
EM-1 Microbial inoculants®	TeraGanix	All types of vegetables and trees	Improved product quality
Bio-Tilis™	Agrovergel	Horticultural crops	Enhanced crop quality

## 5. Conclusions and Future Research Perspectives

Unlike chemical fertilizers, the application of plant probiotic bacteria as green products in the field enhances the nutritional quality of fruits and vegetables. Therefore, the reduced use of chemical fertilizers in favor of products based on probiotic bacteria is a good alternative, not only to reduce the overuse of chemical fertilizer but also to ensure food quality. It has been reported on many occasions how the application of green products enhances crop production, reduces pathogen and pest attacks and increases plant nutrient content. However, to improve the use of biofertilizers many changes need to be made. This includes establishing more effective quality control systems that ensure commercial products are safely made and with nontoxic bacteria. As regards green products need to be designed using suitable carriers that allow these products to be successful in a broad spectrum of environmental conditions.

In this review, we have described several cases of plant probiotic bacteria that when used as biofertilizers act to promoter plant growth, produce increases in yield and improve some parameters associated with food quality. According to some recent studies, bacterial plant probiotics are able to establish a relationship with fruit and horticultural crops and as a result of this interaction some plant compounds, which are beneficial to human health, have an increased production. On one hand, as outlined above, the market for bacterial fertilizers is continuously growing, but the effects of these commercialized bacteria on food quality parameters are not detailed. Therefore, more studies on the effect of improved food quality caused by different types of bacteria are necessary. This would allow for the better selection of plant probiotic bacteria with potential applications in agricultural practices orientated toward the production of high quality fruits and vegetables.

On the other hand, it has been reported how the application of plant probiotic bacteria improve the quality of fruit and vegetables by increasing vitamins, flavonoids and antioxidants content, among other benefits. However, there are other functional food compounds that can also be potentially improved by bacterial inoculants. In the future, new research approaches such as metabolomic studies, comparing fruits and vegetables grown with and without the application of plant probiotic bacteria, may reveal additional beneficial effects on the quality of food crops from the applications of these types of bacteria.
